# Uniformly dispersed ruthenium nanoparticles on porous carbon from coffee waste outperform platinum for hydrogen evolution reaction in alkaline media

**DOI:** 10.1038/s41598-024-56510-7

**Published:** 2024-03-11

**Authors:** Bayaraa Sukhbaatar, Wang Qing, Jinmyeong Seo, Sanghwa Yoon, Bongyoung Yoo

**Affiliations:** Department of Materials Science and Chemical Engineering, Hanyang University, Ansan, 15588 Korea

**Keywords:** Ruthenium nanoparticles, Carbon catalyst, Hydrogen evolution reaction, Spent coffee grounds, HER catalyst, Chemistry, Engineering, Materials science, Nanoscience and technology

## Abstract

Biowaste-derived carbon materials are a sustainable, environmentally friendly, and cost-effective way to create valuable materials. Activated carbon can be a supporting material for electrocatalysts because of its large specific surface area and porosity. However, activated carbon has low catalytic activity and needs to be functionalized with heteroatoms, metals, and combinations to improve conductivity and catalytic activity. Ruthenium (Ru) catalysts have great potential to replace bench market catalysts in hydrogen evolution reaction (HER) applications due to their similar hydrogen bond strength and relatively lower price. This study reports on the synthesis and characterizations of carbon-supported Ru catalysts with large surface areas (~ 1171 m^2^ g^−1^) derived from coffee waste. The uniformly dispersed Ru nanoparticles on the porous carbon has excellent electrocatalytic activity and outperformed the commercial catalyst platinum on carbon (Pt/C) toward the HER. As-synthesized catalyst needed only 27 mV to reach a current density of 10 mA cm^−2^, 58.4 mV dec^−1^ Tafel slope, and excellent long-term stability. Considering these results, the Ru nanoparticles on coffee waste-derived porous carbon can be utilized as excellent material that can replace platinum-based catalysts for the HER and contribute to the development of eco-friendly and low-cost electrocatalyst materials.

## Introduction

According to a statistical report, global coffee production has reached 175 million coffee bags, which are 60 kg, and produce a massive amount of organic waste. One ton of coffee product generates approximately 650 kg of coffee waste, known as spent coffee grounds (SCG), after coffee brewing. It is easy to calculate worldwide annual SCGs, which are more than 6.8 million tons of waste, including fine-sized particles and high humidity, organic load, and acidity^1^. Therefore, sustainable treatment and management solutions are urgently needed to reduce the environmental pollution of SCG and other biowastes. As reported in the literature, coffee waste can be turned into valuable activated carbon and utilized in many applications, such as adsorbents^2,3^, electrocatalysts^4–6^, fuel cells^7,8^, supercapacitors^9,10^, and batteries^11,12^, due to its high specific surface area and porosity. Biowaste-derived activated carbon is a greener pathway for solving pollution issues and provides a demand for sustainability and cost-effectiveness.

The most efficient and widely used HER catalyst is platinum (Pt), but its high cost, scarcity, and poor stability are obstacles to large-scale utilization^13,14^. Thus, Pt-replaceable, effective, and cheaper catalysts, such as Pt group metal-free (PGM-free) carbon or metal-free carbon-based catalysts, have been increasingly reported. However, their HER activity and efficiency cannot outperform those of Pt or Pt-based catalysts, especially in alkaline electrolytes^15–17^. Notably, the alkaline HER requires extra dissociation energy to produce a proton by breaking the O–H–O bond in the Volmer step^18^. Research trends on ruthenium (Ru) catalysts have recently increased due to their similar hydrogen bond strength (~ 65 kcal mol^−1^) to that of Pt and relatively lower price (~ 4% of Pt)^19,20^. Many Ru nanoparticles, Ru single atoms, and carbon-supported Ru catalysts have been introduced for the hydrogen evolution reaction^21–24^. Ma’s research group recently reported a chitin-derived carbon catalyst with a ruthenium catalytic material containing N-doped carbon and Ru nanoparticles due to metal-support interactions; this material has good electrochemical activity and is stable for the HER^25^. Other researchers have developed Ru nanoparticles (NPs) catalysts supported on carbon quantum dots, which are rich in nitrogen and perform an efficient HER catalyst (η_10_ = 65 mV)^26^. Even if the Ru NPs on nitrogen-doped carbon can increase the stability of catalysis, the metallic active site could be blocked by the formation of Ru–N coordinations, reducing HER activity^20,27^. Therefore, it is still difficult to completely control the coordination and surface modification by nitrogen concentration, annealing temperature, etc. As mentioned above, to meet the demands of coffee waste recycling and Pt-replaceable superior active HER catalysts, we aimed to synthesize the uniformly dispersed Ru NP on coffee waste-derived porous carbon and apply it as an electrocatalyst for alkaline HER. There was no additional nitrogen doping to make it easy to control the active sites. However, fewer efforts have been made to use SCG-derived carbon as an electrocatalyst for the HER and other green energy applications^4,28^. Pandley et al. published a study of a porous carbon catalyst from coffee waste, revealing high durability for hydrogen evolution, but the HER activity was not enough to replace commercial catalysts. To our knowledge, no publication on Ru NP catalysts for use as coffee waste-derived carbon supports for HER or even other energy applications has been reported.

In this research, we synthesized an excellent, effective Ru catalyst on a coffee waste-derived porous carbon (Ru@SCC-KU) by a straightforward method in which SCC-KU was impregnated with ruthenium chloride (RuCl_3_·H_2_O) and annealed at high temperature. Ru@SCC-KU was performed as an excellent electrocatalyst for the HER in an alkaline electrolyte (1.0 M NaOH). The uniform dispersion of Ru on porous structured carbon (SCC-KU) has resulted in higher HER activity and greater stability than those of commercial catalysts (Pt/C, 10%). This result suggested that green valorization of coffee waste is a simple strategy for preparing valuable catalysts to replace Pt-based catalysts for the hydrogen evolution reaction.

## Experimental

### Materials

Potassium hydroxide (KOH, 85.0%), urea (CO(NH_2_)_2_, ≥ 98.0%), 1.0 M hydrochloric acid, and 1.0 M NaOH were purchased from Daejung Chemicals. Ruthenium chloride hydrate (RuCl_3_·H_2_O), platinum on graphitized carbon (10 wt% Pt/C), and zinc chloride (ZnCl_2_, ≥ 97.0%) were purchased from Sigma‒Aldrich. Ruthenium on carbon (5 wt%, Ru/C) and 1,10-phenanthroline monohydrate (C_12_H_8_N_2_·H_2_O, > 98%) were obtained from the Tokyo chemical industry. Nafion polymer dispersion (D521, 5 wt%) was purchased from Chemours. Carbon black (Super P^®^ conductive, > 99%) was obtained from Thermo Scientific. Spent coffee grounds (SCGs) were collected after coffee brewing Starbucks (medium roast).

## Methods

### Synthesis of SCC-KU

Coffee waste-derived carbon (SCC-KU) was synthesized by a method similar to that used in our previous work^29^. Briefly, powdered SCG was washed with 1.0 M HCl several times to remove impurities and dried. Then, after mixing with KOH and urea, 50 ml of distilled water was added, the mixture was agitated for 12 h and dried. Finally, the mixture was pyrolyzed in a furnace tube with nitrogen gas flowing at 500 mL min^−1^ at 800 °C for 2 h. After cooling under nitrogen flow, the sample was leached with 1.0 M hydrochloric acid, rinsed with distilled water until neutral, and dried at 105 °C for 12 h.

### Synthesis of the Ru@SCC-KU catalyst

**Figure 1:**
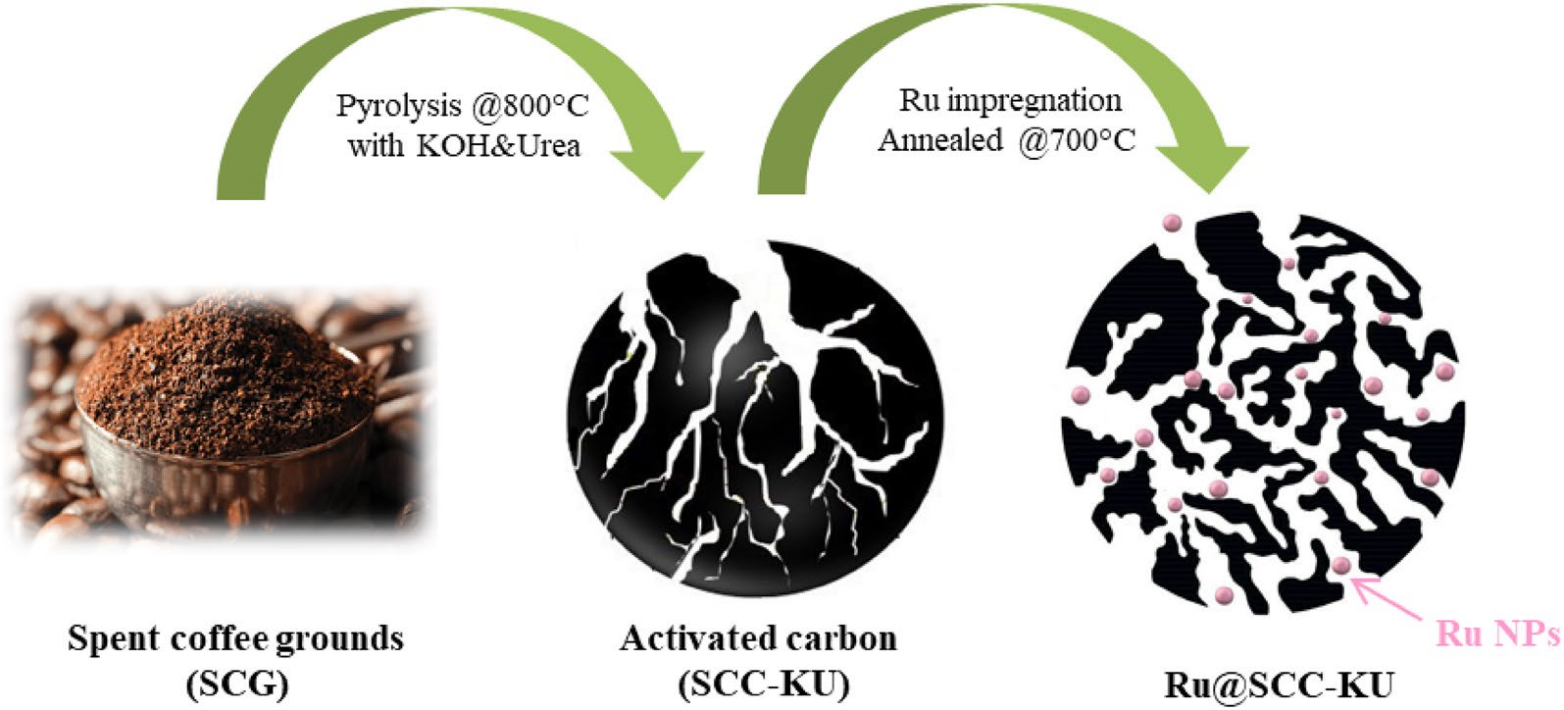
Diagrammatic illustration of the Ru@SCC-KU synthesis.

SCC-KU was used as a carbon support for further preparation of electrocatalysts. First, a 10 mg mL^−1^ ruthenium chloride hydrate solution was prepared in ethanol as a stock solution. Next, 2.08 mL (equivalent to 0.1 mmol, 20.74 mg of RuCl_3_·H_2_O) of stock solution was placed into the tube with 2.0 mL of ethanol. Then, 100 mg of SCC-KU was added, sonicated for 30 min, and agitated for 12 h under a magnetic stirrer (Ru-impregnated carbon). Finally, the mixture was dried at 110 °C, mixed well with a mortar and pestle, and annealed under nitrogen gas flow rate of 500 mL min^−1^ at 700 °C for 2 h, as illustrated in Fig. 1. As a result, the synthesized catalyst is denoted as Ru@SCC-KU.

For comparative studies, other samples were prepared by the same method. Ru@SCG-KU-8 and Ru@SCC-KU-6 were annealed at 800 °C and 600 °C, respectively. Ru@CB and Ru@SCC-Z were prepared using commercial carbon black and ZnCl_2_-activated carbon. More details on the comparative sample preparations are available in the Supplementary information.

### Materials characterization

The surface morphologies were examined via field emission scanning electron microscopy (FE-SEM) (TESCAN MIRA3), and transmission electron microscopy (TEM) images were obtained at 200 keV (JEOL, JEM-2100F). A nitrogen sorption analyzer was used to determine the surface area and pore distribution of the as-prepared samples at 77 K (TrisStar II 3020). X-ray photoelectron spectroscopy (XPS) was performed with a Thermo Fisher instrument (NEXSA), and X-ray diffraction (XRD) was performed on a Bruker (D8 Advance, using Cu Ka radiation, λ = 1.5 Å). Raman analysis was performed using a Thermo Fisher (DXR3xi) micro-Raman spectrometer with a laser wavelength of 532 nm.

### Electrocatalytic HER performance

Electrochemical analyses were carried out using a potentiostat (VersaSTAT 4) with a typical three-electrode system and a rotating disk electrode (RDE) (Pine Research MSR Rotator). The temperature was controlled by circulating water at 25 °C. A 1.0 M NaOH electrolyte was used for all the electrochemical tests. A 5 mm glassy carbon electrode (GCE) and a graphite rod were used as the working and counter electrodes. The alkaline mercury/mercurous oxide (Hg/HgO, 1.0 M NaOH) was used as a reference electrode.

All the measured potential values were converted to a reversible hydrogen electrode (RHE) according to the reference electrode calibration and Eq. (1).

1 $$E_{RHE} = \left( {E_{Hg/HgO} + 0.0591 pH + 0.14} \right) = E_{Hg/HgO} + 0.926$$

Linear sweep voltammetry (LSV) was performed with a scan rate of 5 mV s^−1^ and potentials between 0 and − 0.40 V (vs RHE). Electrochemical impedance spectroscopy (EIS) analysis was performed at 0.01 to 10^6^ Hz at a constant voltage of − 0.01 V (vs RHE). The double layer capacitance was determined using cyclic voltammetry (CV) at different scan rates (5, 10, 20, 40, and 60 mV s^−1^) in the nonfaradaic region (from 0.1 to 0.2 V vs RHE). The long-term stability of the catalysts was tested by chronopotentiometry (CP) at 10 mA cm^−2^ for 24 h. Compensation (iR) was not applied in any of the electrochemical measurements.

The working electrodes were prepared by the simple drop-casting method. First, 5 mg of catalyst was uniformly dispersed in 0.90 μL of 40% isopropyl alcohol with deionized (DI) water and ultrasonicated for 30 min. Then, 10.0 μL of 5 wt% Nafion solution was added and mixed. The mixture was agitated on a magnetic stirrer for 12 h. Finally, a total of 24 μL (by 8.0 μL, three times) of catalyst ink was drop cast onto a 5 mm glassy carbon electrode (GCE) and dried at room temperature (loading mass: ∼0.612 mg cm^−2^).

## Results and discussion

### Structural characterization

**Figure 2:**
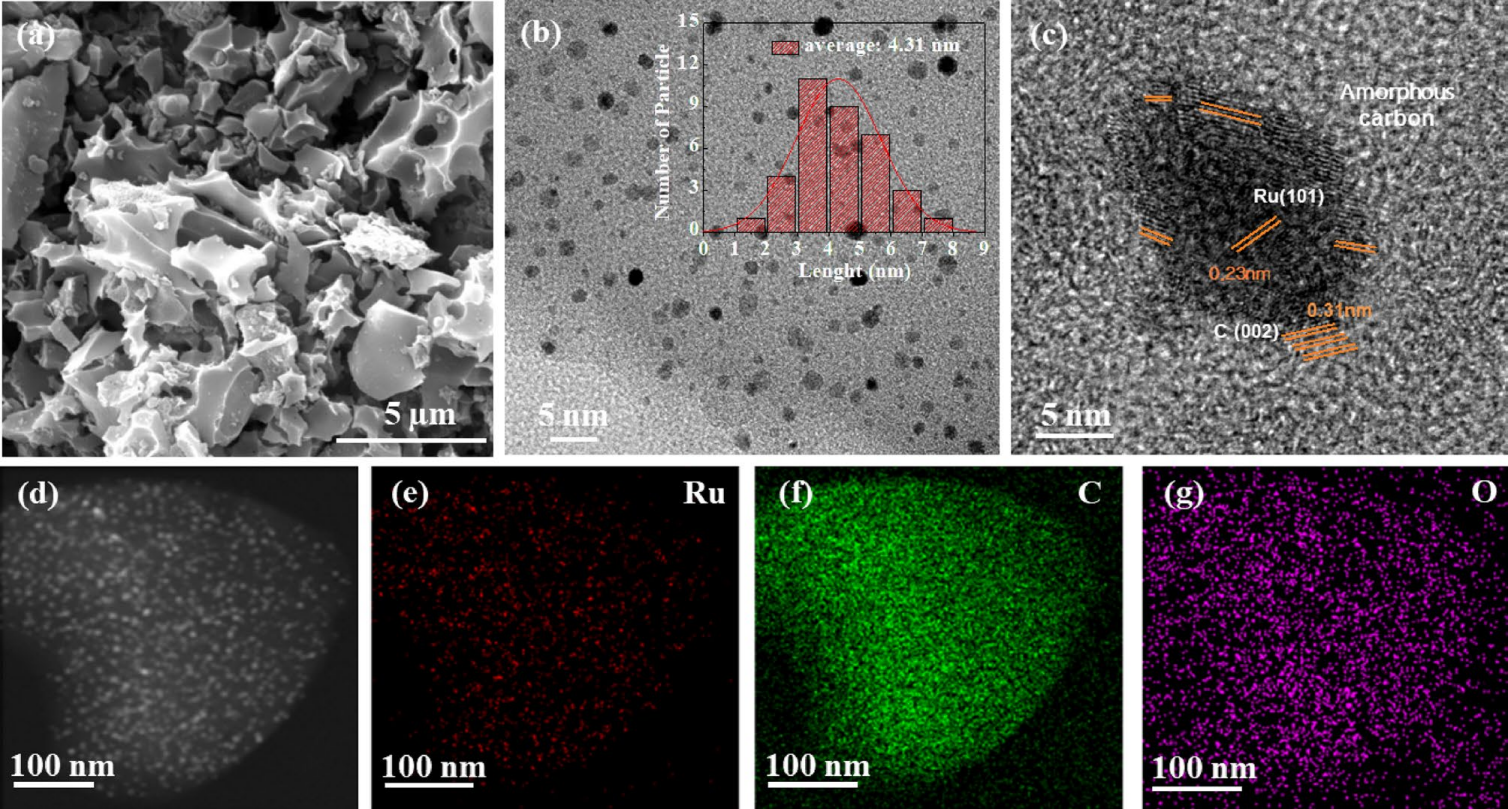
(**a**) FE-SEM image of Ru@SCC-KU. (**b**) TEM image of Ru@SCC-KU (inset Ru NPs dispersion). (**c**) HRTEM image of Ru@SCC-KU. (**d**) HAADF-STEM image of Ru@SCC-KU with (**e**, **f**, **g**) corresponding elemental mappings Ru, C, and O.

The catalyst preparation and synthesis steps are illustrated in Fig. 1. Spent coffee ground-derived carbon (SCC-KU) was synthesized by pyrolysis with KOH and urea activation at 800 °C. SCC-KU cells were impregnated with ruthenium chloride and annealed at 700 °C (Ru@SCC-KU). As shown in Fig. 2a, the FE-SEM image revealed the hierarchical porous and interconnected morphology of Ru@SCC-KU, which may result in a high surface area and facile uniform dispersion of ruthenium nanoparticles (Ru NPs). No significant differences were observed between SCC-KU and Ru@SCC-KU, as shown in Fig. S1.

TEM was used to evaluate the particle size and specific morphology of the Ru NPs on the electrocatalysts. As shown in Fig. 2b, the TEM image displays the uniform distribution of Ru NPs on the carbon support. HR-TEM images revealed the presence of spherical Ru NPs distributed on the porous carbon texture, which formed particles with an average size of 4.31 nm. The *d*-spacing of the Ru NPs was determined to be 0.23 nm (Fig. 2c). The HAADF-STEM image is shown in Fig. 2d, and elemental mapping images are shown in Fig. 2e, f, and g, corresponding to the uniform dispersion of Ru, C, and O on the Ru@SCC-KU. TEM images of Ru@SCC-KU-6 and Ru@SCC-KU-8 revealed similar results to those for Ru@SCC-KU, but some agglomerations of NPs were observed for Ru@SCC-KU-6 (Fig. S2). Ru@SCC-KU-U and Ru@SCC-KU-N3 show poor uniform dispersion of Ru on the porous carbon support. Ru@CB has an onion-like amorphous carbon structure and more and fewer dots than the other materials^1,30^.

**Figure 3:**
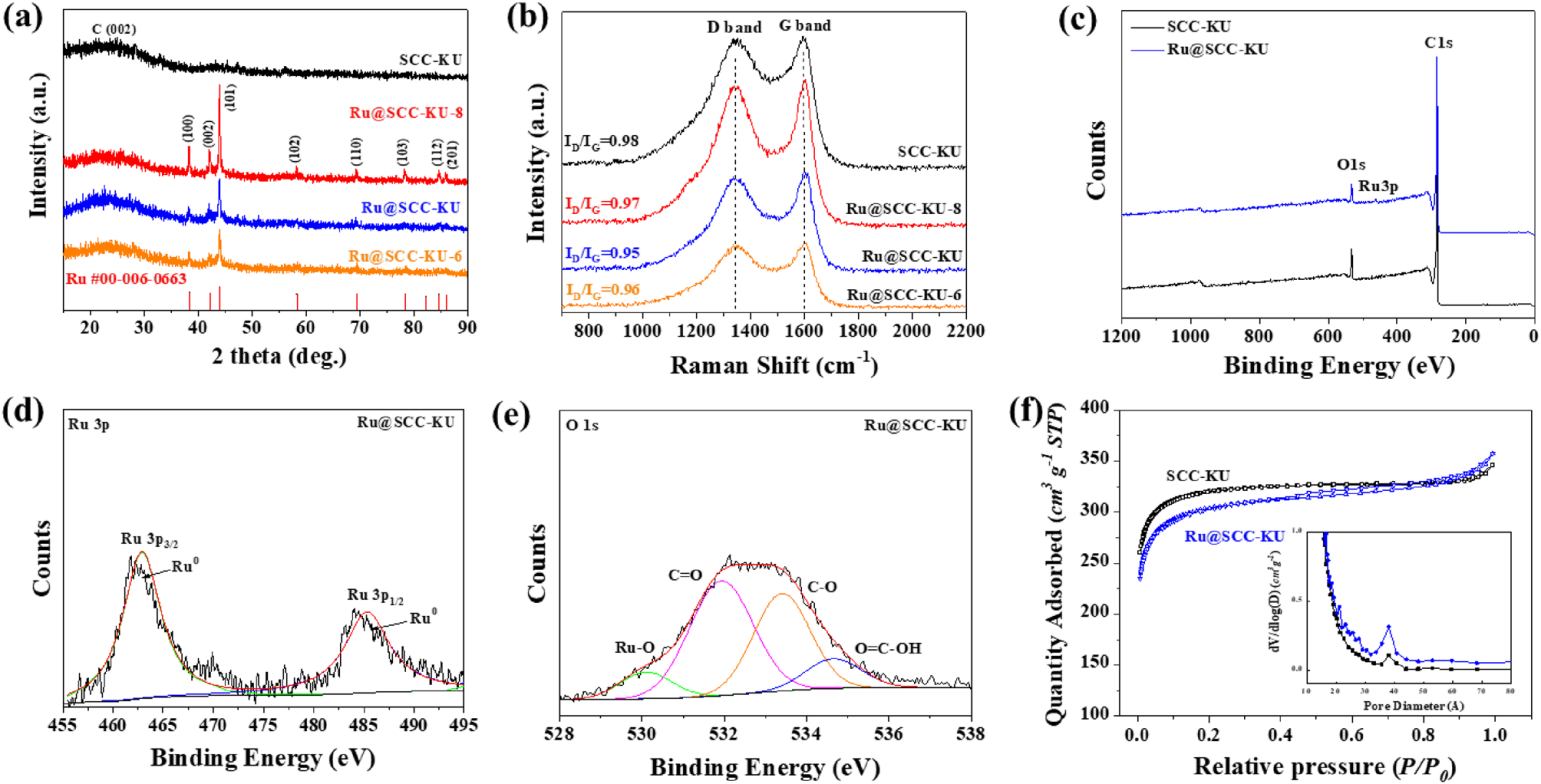
(**a**) XRD patterns, (**b**) Raman spectra, and (**c**) XPS survey spectra of electrocatalysts. High-resolution XPS spectra of (d) Ru3p and (**e**) O1s of Ru@SCC-KU. (**f**) N_2_ adsorption and desorption isotherms (inset pore size distributions) of the prepared catalysts.

XRD and Raman spectroscopy were carried out to test the electrocatalyst properties. The XRD patterns of SCC-KU, Ru@SCG-KU, Ru@SCG-KU-8, and Ru@SCG-KU-6 are shown in Fig. 3a**.** Generally, broad peaks corresponding to planar graphite (002) and hexagonal structured carbon (100) structures are detected at 23° and 43°, respectively, in the spectra of the samples^10,31^. The sharpest diffraction peaks related to metallic ruthenium (Ru^0^) (JCPDS No 00-006-0663) were clearly observed, validating the existence of Ru NPs. The sharp peaks centered at 38.4, 42.1, 44.0, 58.3, 69.4, 78.4, 84.7, and 85.9° indicate the lattice planes of Ru^0^, corresponding to (100), (002), (101), (102), (110), (103), (112), and (201), respectively. The high-temperature annealed catalyst (Ru@SCC-KU-8) is sharper than the others, indicating more crystallinity, which validates the HR-TEM results. The peak intensity of Ru@SCC-KU annealed at 700 °C was lower than that of Ru@SCC-KU annealed at high temperature, indicating the formation of Ru clusters at higher temperatures^32^. The XRD patterns of the other catalysts are displayed in Fig. S3. The spectra of the catalysts revealed similar Ru peaks and general broad peaks for amorphous carbon structures. According to the TEM image results, Ru@CB and commercial carbon black exhibited more intense XRD peaks, confirming the existence of a defective structure rather than as-prepared coffee waste-derived carbons^33^. The commercial catalyst (Ru/C) may contain a more crystalline carbon structure, showing a sharp peak around 27°, and may come from oxidized ruthenium (RuO_2_), such as RuO_2_ (JCPDS No 01-088-0322)^22^.

In Raman spectroscopy (Fig. 3b), two distinguishable peaks are centered at 1343 cm^−1^ (D band) and 1600 cm^−1^ (G band). The intensity ratio (*I*_*D*_*/I*_*G*_) of the D band (*sp*3) to the G band (*sp*2) is a standard method for estimating the graphitic degree and defects of the carbon lattice^34^. The intensity ratios (*I*_*D*_*/I*_*G*_) for SCC-KU, Ru@SCC-KU-8, Ru@SCC-KU-6, and Ru@SCC-KU were calculated to be 0.98, 0.97, 0.96, and 0.95, respectively, indicating that the Ru@SCC-KU catalysts have structured more crystalline than the parent carbon support (SCC-KU) due to high-temperature annealing and ruthenium impregnation. Additionally, broad peaks could be observed at approximately 1450 cm^−1^ and 1150 cm^−1^, corresponding to the presence of amorphous carbon and the coexistence of *sp*3 and *sp*2 hybridized carbon phases, respectively^35^. The Raman shifts of the other comparative catalysts displayed higher intensity ratios (*I*_*D*_*/I*_*G*_), as shown in Fig. S4, which may be attributed to the abundance of defects in the carbon framework. These results were in excellent agreement with the TEM and XRD analyses.

Moreover, X-ray photoelectron spectroscopy (XPS) was carried out to estimate the chemical components and oxidation states of the elements in the catalysts. As shown in Fig. 3c, the XPS survey spectrum revealed the presence of metallic Ru, oxygen, and carbon in the Ru@SCC-KU cells. The Ru3p high-resolution spectrum exhibited two peaks at 463.7 and 485.0 eV attributed to metallic ruthenium (Ru^0^), as indicated by Ru3p_2/3_ and Ru3p_1/3_ (Fig. 3d), confirming the absence of an apparent oxidation state in the Ru NPs^22^. In contrast, Ru@SCC-KU-U and Ru@SCC-KU-N3 exhibited two pairs of peaks corresponding to Ru^0^ and oxidized Ru (Fig. S5), which can be effects of additional nitrogen dopants, which can form Ru–N bonds in the carbon network. The high-resolution spectrum of C1s is shown in Fig. S6. The peaks at approximately 284.7, 286.2, 287.8, 289.7, and 291.8 eV were attributed to the C=C/C–C, C–O, C=O, C=O–C, and π − π^*^ satellites, respectively, which is in good agreement with the coexistence of *sp*3 and *sp*2 hybridized carbon atoms and π − π^*^ satellites and reconfirms the presence of C=C bonds^36^. The high-resolution O1s spectrum confirmed that Ru@SCC-KU exhibited significant peaks corresponding to C=O, C–O, and O=C–OH (Fig. 3e) and even to trace amounts of Ru–O, which may be attributed to Ru–O–C interactions due to the presence of oxygen functional groups in the carbon texture. This result is significant evidence of Ru–O–C bonding during the impregnation process because parent carbon (SCC-KU) is rich in oxygen functional groups. As comparative catalysts, Ru@SCC-KU-U and Ru@SCC-KU-N3 possess Ru–O, C–O, and C=O bonded structures (Fig. S7). Hence, the XPS analysis confirmed that the metallic Ru NPs on Ru@SCC-KU formed a strong bond (Ru–O–C) in the porous and amorphous carbon network, as indicated by the XRD and Raman results. According to the XPS analysis, Ru@SCC-KU contains negligible (0.49 at%) amounts of ruthenium and 94.5 and 5.01 at% C and O, respectively (Table S1). The Ru content was similar to that determined via ICP analysis (3.90 wt%) if converted to the value (at%).

Interestingly, the oxygen content decreased significantly (SCC-KU: 7.33 at% of O1s) due to the reaction with metal moieties during impregnation and annealing. Because of the optimal size of defects, oxygen functional groups on the carbon surface, and high surface area, SCC-KU may have a high adsorption capacity for metal cations in solution^37^. This may be the critical reason for the uniform dispersion and strong interaction of Ru–O–C in the as-synthesized Ru@SCC-KU catalyst. Such the formation of Ru–O–C networks can enhance the number of electrocatalytic active sites and their conductivity, preventing the aggregation of NPs, as reported in recent publications^22,24,38^. The components of Ru@SCC-KU-6 are similar to our focused catalyst Ru@SCC-KU. However, according to the XRD results, Ru@SCC-KU-8 has slightly greater Ru (0.51 at%) and lower oxygen (4.19 at%) contents than the other samples. Ru@SCC-KU-U and Ru@SCC-KU-N3 had lower carbon contents and higher oxygen, Ru, and nitrogen contents than the other nitrogen-added samples. According to the literature, the combination of nitrogen, carbon, and metallic Ru could be an efficient and high-durability catalyst for HER application by facilitating suitable interactions between Me and N–C^4,39,40^.

Nitrogen adsorption and desorption isotherms were used to study the specific surface area and pore structure of the electrocatalysts. As shown in Fig. 3f, SCC-KU has no hysteresis loops, similar to type I isotherms, which may have a micropore-dominated structure^41^. In contrast, Ru@SCC-KU exhibited a type IV isotherm with a hysteresis loop between 0.4 and 0.8 *P/P*_0_, indicating mesopore formation in the carbon texture^42^. Ru@SCC-KU has a greater mesopore volume than the other comparative samples (Fig. 3f, inset), which validates the enlargement of pore size and volume after Ru impregnation and annealing. Ru@SCC-KU and the parent carbon SCC-KU possess large BET surface areas of 1170.9 and 1243.9 m^2^ g^−**1**^, respectively, which are slightly reduced after annealing (Table S2). These results also confirm that the formation of Ru NPs did not block carbon pores and maintained a larger surface area. Thus, Ru@SCC-KU-N3 has a lower surface area and poor pore volume, which may be attributed to obstacles from the Ru–N complex and Ru NP clusters and the blockage of a porous morphology during the impregnation procedure. Hence, the increased specific surface area of the as-synthesized porous carbon (SCC-KU) could increase the number of Ru NP active sites and their conductivity by preventing the aggregation of metal species and providing a uniform distribution in the hierarchical porous structure^43^. Several efforts have been made to improve the HER activity of ruthenium catalysts on porous carbon supports^19,22–24^; however, few attempts have been made to use biowaste-derived carbon as a support^20,25^.

### Electrochemical analysis

**Figure 4:**
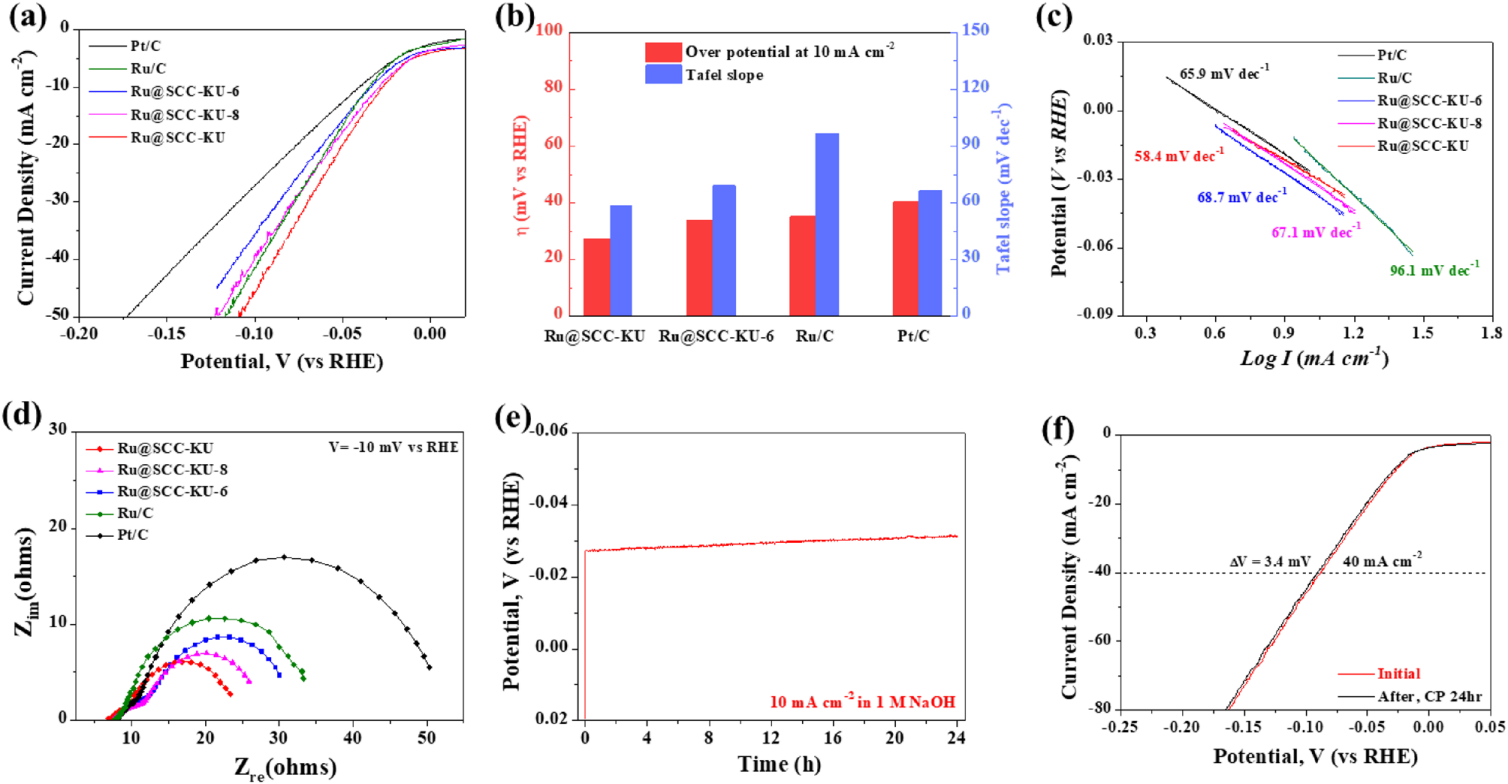
(**a**) Linear sweep voltammetry (LSV) curves of HER in the alkaline medium (1 M NaOH), (**b**) the diagram of the overpotentials at the 10 mA cm^−2^ and Tafel slopes. (**c**) Tafel curves obtained from LSV of Pt/C 10%, Ru/C 5%, Ru@SCC-KU-6, Ru@SCC-KU-8, and Ru@SCC-KU. (**d**) Nyquist plots of samples at an overpotential of − 10 mV, (**e**) chronopotentionmetric curve of Ru@SCC-KU recorded at 10 mA cm^−2^ of current density for 24 h, and (**f**) the long-term stability of Ru@SCC-KU in alkaline electrolyte.

The electrocatalytic activity of the as-synthesized samples was examined through a three-electrode system in N_2_-saturated 1.0 M NaOH electrolyte at a constant 25 °C. The mass loading of the catalyst on the glassy carbon was ~ 0.612 mg cm^−2^. Figure 4a shows linear sweep voltammetry (LSV) plots at a scan rate of 5 mV s^−1^ and 1600 rpm with a rotating disk electrode (RDE) without iR compensation for the Ru@SCC-KU and control samples. We investigated the electrocatalytic activity of bench marketing catalysts, such as Pt/C (10 wt% platinum on graphitized carbon) and Ru/C (5 wt% ruthenium on carbon), as a reference. The Hg/HgO and NaOH (1.0 M) reference electrodes were calibrated against a reversible hydrogen electrode (RHE) (Fig. S8, Supplementary Note 1). The overpotential (η_10_) at a current density of 10 mA cm^−2^ is used as a standard evaluation parameter for electrocatalysts because it is equivalent to the 12.3% efficiency of a solar water-splitting device^44^. As expected, Pt/C and Ru/C exhibited high HER activities that needed 40.1 and 35.1 mV, respectively, to reach a current density of 10 mA cm^−2^. Ru@SCC-KU has excellent HER activity, outperforming commercial Pt/C and Ru/C, which have the lowest potential (η_10_ = 27.0 mV). Ru@SCC-KU-6 and Ru@SCC-KU-8 also outperformed Pt/C, showing η_10_ = 33.5 mV and η_10_ = 29.2 mV, respectively. This result is in good agreement with the XRD and Raman spectral results. Figure 4b summarizes the overpotentials and Tafel slopes of the as-synthesized and commercial catalysts, in which Ru@SCC-KU has better HER activity than the other materials. According to the Tafel equation, Tafel slopes were calculated from LSV polarizations^45^. As shown in Fig. 4c, Ru@SCC-KU had the lowest Tafel slope (58.4 mV dec^−1^) of any other catalysts. According to the lowest Tafel slope of Ru@SCC-KU, hydrogen evolution reaction may occured by Volmer-Heyrovsky reaction pathway (H_2_O + e^−^  → OH^−^  + H_ad_; H_ad_ + H_2_O + e^−^  → OH^−^  + H_2_)^18,46^. In which, electrolysis reaction is fast due to high hydrogen bond strength (~ 65 kcal mol^−1^) of ruthenium.

The LSV curves and Tafel slopes of the other comparative catalysts are shown in Fig. S9. Interestingly, comparative catalysts, such as Ru@SCC-KU-U (η_10_ = 35.5 mV, Tafel slope: 64.2 mV dec^−1^) and Ru@SCC-Z (η_10_ = 33.5, Tafel slope: 62.3 mV dec^−1^), exhibited good HER performance; these catalysts have higher activity than Pt/C but lower activity than Ru@SCC-KU. These results are related to the high specific surface area and ruthenium active sites of these catalysts. Ru@SCC-KU-N3 (η_10_ = 58.4 mV, Tafel slope: 64.2 mV dec^−1^) and Ru@CB (η_10_ = 99.4 mV, Tafel slope: 101.9 mV dec^−1^) showed poorer HER activity than the other materials. This result is similar to the research of Baek et al*.* on the outstanding HER activity of Ru NPs on graphene nanoplates^47^. However, in the case of Ru on nitrogen-doped support, the catalytic activity was significantly reduced because the active sites of the metal species may be blocked by Ru–N–C coordination^27^. The HER performance of carbon-based catalysts in alkaline electrolytes is summarized in Table S3 to compare our results^22–26,48–53^. This table shows that our results are better than or similar to those of previously reported catalysts. Here, Yuan’s research group reported the use of ruthenium oxide on a nitrogen-doped carbon matrix (RuO_2_/N–C), which exhibited good HER performance (η_10_ = 40 mV)^52^. As mentioned above, because Ru–N coordination may block some active sites on RuNPs, the RuO_2_/N–C catalyst exhibited lower activity than our synthesized catalyst (Ru@SCC-KU).

Electrochemical impedance spectroscopy (EIS) measurements revealed a lower charge transfer resistance and a smaller radius in the Nyquist curve of Ru@SCC-KU than in the other reference catalysts, indicating a faster charge during electrochemical cycling and superior electrode kinetics (Fig. 4d)^22,54^. The Nyquist plots were fitted to an equivalent circuit: R_1_(Q(R_2_(CR_3_))), as shown in Fig. S10a. R_1_ may correspond to solution resistance, R_2_ electrolyte, intermediate layer resistance, where charge transfer occurs, Q constant phase element, C capacitance, and R_3_ is the resistance of the barrier layer. Ru@SCC-KU has a substantially lower charge transfer resistance (R_2_) value (7.56 Ω) than the other samples, including Ru@SCC-KU-8 (10.35 Ω), Ru@SCC-KU-6 (8.83 Ω), Ru/C (11.43 Ω), and Pt/C (12.06 Ω). This lower resistance results from the favorable electron transfer between Ru and the carbon surface, which enables faster charge transfer and improved intrinsic catalytic activity. Additionally, the Nyquist curve radius of the catalysts increased in the following order: Ru@SCC-KU < Ru@SCC-KU-8 < Ru@SCC-KU-6 < Ru/C < Pt/C. As estimated before, the electrochemical activity significantly increased because of the uniform distribution of Ru NPs on the hierarchically porous carbon, which could increase the conductivity.

We determined the turnover frequency (TOF) of Ru@SCC-KU and commercial catalysts to determine the inherent electrocatalytic efficiency following previously reported methods (Supplementary Note 2)^48,55^. The TOF of Ru@SCC-KU was greater (Fig. S10b) than commercial catalysts (Pt/C and Ru/C). Precisely, the TOF of Ru@SCC-KU was estimated to be 0.44 H_2_ s^−1^ at 50 mV, which was higher than that of Ru/C (0.33 H_2_ s^−1^) and Pt/C (0.38 H_2_ s^−1^). These values confirm that Ru@SCC-KU is an excellent commercial catalyst and competitive HER activity with other reported electrocatalysts^38,56^.

Thiocyanate (SCN^−^) and ethylenediaminetetraacetic acid (EDTA) poisoning experiments were employed to determine the main catalytically active species in Ru@SCC-KU cells. EDTA is a complexing agent that selectively coordinates single atomic metal species. Moreover, thiocyanate can form complexes with single atoms and nanoparticles^57^. The poisoning concentration was 50 mM in 1.0 M NaOH electrolyte. As shown in the LSV curves (Fig. S11), there was almost no change after adding EDTA to the electrolyte compared with when no poisoning occurred. The HER catalytic activity decreases significantly in the case of thiocyanate poisoning, suggesting the presence of abundant nanoparticles in the catalyst. Thus, the results validate that Ru NPs are the dominant component as well as the main active sites for HER activity and agree with the previously discussed characterization analysis.

The electrochemical active surface area (ECSA) was tested by cyclic voltammetry (CV) at different scan rates to determine the specific HER activity of the catalyst (Fig. S12). The SCC-KU device had a lower capacitance, with a value of 55.1 mF cm^−2^. The *C*_*dl*_ value of Ru@SCC-KU was estimated to be 70.3 mF cm^−2^, indicating the highest ECSA, which is in good agreement with the finding that porous carbon with Ru NPs could have exposed active sites in the catalyst^58^. In contrast, the capacitance of Pt/C (34.8 mF cm^−2^) was lower than that of SCC-KU. Additionally, the ECSA and active site density of Ru@SCC-KU were calculated with ~ 4.34 10^13^ sites per cm^2^ (Supplementary Note 3)^59^.

A cyclopotentiometric (CP) test was employed for 24 h at 10 mA cm^−2^ to evaluate the long-term stability of Ru@SCC-KU in an alkaline electrolyte. Figure 4e shows that, compared with Pt/C and other catalysts, Ru@SCC-KU exhibited excellent stability, with a slight decrease of ~ 5.2% after a 24-h reaction (Fig. S13a). Figure 4f shows the LSV curves of Ru@SCC-KU before and after the CP test; there was almost no apparent loss, as it displayed only a 3.4 mV negative shift at 40 mA cm^−2^, which confirmed its high stability in an alkaline medium. In addition, after the long-term stability test, TEM images of the Ru@SCC-KU cells exhibited no significant differences in morphology (Fig. S14).

Moreover, acidic leaching is performed on the Ru@SCC-KU catalyst to check durability and recycling availability. After long-term stability testing, Ru@SCC-KU was leached into a 1.0 M HCl solution with constant stirring for 12 h and washed with distilled water until the sample was neutral. Then, Ru@CC-KU was dried at 105 °C for 12 h. Finally, the amount of leached Ru@SCC-KU was checked via an electrochemical test. Surprisingly, there were no apparent differences, such as a negligible decrease in the LSV curves (Fig. S13b) or no morphological changes in the TEM image (Fig. S14c). These results validate the superior stability, durability, and recyclability of Ru@SCC-KU compared to those of commercial catalysts.

## Conclusions

We presented coffee waste conversion to the valuable HER catalyst via simple adsorption and pyrolysis techniques. As-synthesized, Ru@SCC-KU employed excellent HER activity and had high long-term stability in an alkaline medium. Ru@SCG-KU required only 27.0 mV to reach a current density of 10 mA cm^−2^, a high Tafel slope (58.4 mV dec^−1^) and a TOF (0.44 H_2_ s^−1^), outperforming commercial Pt/C and Ru/C catalysts. The superior HER performance of Ru@SCC-KU may be related to the uniformly distributed Ru NPs enhancing the number of active sites, the suitable Ru–O–C interactions in the highly porous carbon framework, and the high surface area. Most importantly, Ru@SCC-KU is a straightforward and low-cost carbon precursor amenable to green engineering and biowaste waste management, making it advantageous for use as an excellent electrocatalyst.

### Supplementary Information

Supplementary Information.

## Data Availability

All data generated and analysed during this study, which include experimental, spectroscopic, crystallographic, and computational data, are included in this article and its supplementary information. Further data is available from the corresponding author on reasonable request.

## References

[1] Colantoni A (2021). Spent coffee ground characterization, pelletization test and emissions assessment in the combustion process. Sci. Rep..

[2] Oliveira WE, Franca AS, Oliveira LS, Rocha SD (2008). Untreated coffee husks as biosorbents for the removal of heavy metals from aqueous solutions. J. Hazard. Mater..

[3] Wen X (2019). Large-scale converting waste coffee grounds into functional carbon materials as high-efficient adsorbent for organic dyes. Bioresour. Technol..

[4] Srinu A, Peera SG, Parthiban V, Bhuvaneshwari B, Sahu AK (2018). Heteroatom engineering and Co–doping of N and P to porous carbon derived from spent coffee grounds as an efficient electrocatalyst for oxygen reduction reactions in alkaline medium. ChemistrySelect.

[5] Stock S (2022). Coffee waste-derived nanoporous carbons for hydrogen storage. ACS Appl. Energy Mater..

[6] Chen Z (2022). Waste-derived catalysts for water electrolysis: Circular economy-driven sustainable green hydrogen energy. Nano-Micro Lett..

[7] Jang H, Ocon JD, Lee S, Lee JK, Lee J (2015). Direct power generation from waste coffee grounds in a biomass fuel cell. J. Power Sour..

[8] Kaya M (2020). Evaluating organic waste sources (spent coffee ground) as metal-free catalyst for hydrogen generation by the methanolysis of sodium borohydride. Int. J. Hydrog. Energy.

[9] Choi J (2019). Waste coffee management: Deriving high-performance supercapacitors using nitrogen-doped coffee-derived carbon. C.

[10] Adan-Mas A, Alcaraz L, Arévalo-Cid P, López-Gómez FA, Montemor F (2021). Coffee-derived activated carbon from second biowaste for supercapacitor applications. Waste Manag..

[11] Krikstolaityte V (2018). Conversion of spent coffee beans to electrode material for vanadium redox flow batteries. Batteries.

[12] Xie Q, Qu S, Zhang Y, Zhao P (2021). Nitrogen-enriched graphene-like carbon architecture with tunable porosity derived from coffee ground as high performance anodes for lithium ion batteries. Appl. Surf. Sci..

[13] Liu H (2017). Uniformly dispersed platinum-cobalt alloy nanoparticles with stable compositions on carbon substrates for methanol oxidation reaction. Sci. Rep..

[14] Zhang T (2022). Pinpointing the axial ligand effect on platinum single-atom-catalyst towards efficient alkaline hydrogen evolution reaction. Nat. Commun..

[15] Jia G (2021). Urea-assisted synthesis and tailoring cobalt cores for synergetic promotion of hydrogen evolution reaction in acid and alkaline media. Adv. Energy Sustain. Res..

[16] Muhyuddin M (2022). Valorization of the inedible pistachio shells into nanoscale transition metal and nitrogen codoped carbon-based electrocatalysts for hydrogen evolution reaction and oxygen reduction reaction. Mater. Renew. Sustain. Energy.

[17] Sun Y (2021). Biomass-derived nitrogen self-doped porous activation carbon as an effective bifunctional electrocatalysts. Chin. Chem. Lett..

[18] Mahmood N (2018). Electrocatalysts for hydrogen evolution in alkaline electrolytes: Mechanisms, challenges, and prospective solutions. Adv. Sci..

[19] Bae S-Y, Mahmood J, Jeon I-Y, Baek J-B (2020). Recent advances in ruthenium-based electrocatalysts for the hydrogen evolution reaction. Nanoscale Horiz..

[20] Luo W, Wang Y, Cheng C (2020). Ru-based electrocatalysts for hydrogen evolution reaction: Recent research advances and perspectives. Mater. Today Phys..

[21] Bat-Erdene M (2021). Highly dispersed Ru nanoparticles on boron-doped Ti_3_C_2_T_x_ (MXene) nanosheets for synergistic enhancement of electrocatalytic hydrogen evolution. Small.

[22] He Q (2022). Synergic reaction kinetics over adjacent ruthenium sites for superb hydrogen generation in alkaline media. Adv. Mater..

[23] Kweon DH (2020). Ruthenium anchored on carbon nanotube electrocatalyst for hydrogen production with enhanced Faradaic efficiency. Nat. Commun..

[24] Yang Y (2022). Bridge the activity and durability of Ruthenium for hydrogen evolution reaction with the RuOC link. Chem. Eng. J..

[25] Ma E (2022). Chitin derived carbon anchored ultrafine Ru nanoparticles for efficient hydrogen evolution reaction. ACS Sustain. Chem. Eng..

[26] Li W (2020). Carbon quantum dots enhanced the activity for the hydrogen evolution reaction in ruthenium-based electrocatalysts. Mater. Chem. Front..

[27] Wang J, Wei Z, Mao S, Li H, Wang Y (2018). Highly uniform Ru nanoparticles over N-doped carbon: pH and temperature-universal hydrogen release from water reduction. Energy Environ. Sci..

[28] Akula S, Sahu AK (2019). Heteroatoms co-doping (N, F) to the porous carbon derived from spent coffee grounds as an effective catalyst for oxygen reduction reaction in polymer electrolyte fuel cells. J. Electrochem. Soc..

[29] Sukhbaatar B, Yoo B, Lim J-H (2021). Metal-free high-adsorption-capacity adsorbent derived from spent coffee grounds for methylene blue. RSC Adv..

[30] Zheng T (2019). Large-scale and highly selective CO_2_ electrocatalytic reduction on nickel single-atom catalyst. Joule.

[31] Yun YS (2015). Hierarchically porous carbon nanosheets from waste coffee grounds for supercapacitors. ACS Appl. Mater. Interfaces.

[32] Yang H (2019). A universal ligand mediated method for large scale synthesis of transition metal single atom catalysts. Nat. Commun..

[33] Kamińska, A. *et al.* Activated carbons obtained from orange peels, coffee grounds, and sunflower husks&mdash;comparison of physicochemical properties and activity in the alpha-pinene isomerization process. *Materials* **14** (2021). 10.3390/ma14237448 PMC8659265 34885604

[34] Ferrari AC, Robertson J (2000). Interpretation of Raman spectra of disordered and amorphous carbon. Phys. Rev. B.

[35] Sarathchandran, C., Devika, M. R., Prakash, S., Sujatha, S. & Ilangovan, S. A. In *Handbook of Carbon-Based Nanomaterials* (eds Sabu Thomas, C. Sarathchandran, S. A. Ilangovan, & Juan Carlos Moreno-Piraján) 783–827 (Elsevier, 2021).

[36] Bansal, R. C. & Goyal, M. In *Activated Carbon Adsorption* Ch. 1, 1–58 (Taylor & Francis, 2005).

[37] Wang B, Lan J, Bo C, Gong B, Ou J (2023). Adsorption of heavy metal onto biomass-derived activated carbon: Review. RSC Adv..

[38] Luo D (2018). Biomimetic organization of a ruthenium-doped collagen-based carbon scaffold for hydrogen evolution. J. Mater. Chem. A.

[39] Prabu N, Kesavan T, Maduraiveeran G, Sasidharan M (2019). Bio-derived nanoporous activated carbon sheets as electrocatalyst for enhanced electrochemical water splitting. Int. J. Hydrog. Energy.

[40] Tai S-H, Chang BK (2019). Effect of nitrogen-doping configuration in graphene on the oxygen reduction reaction. RSC Adv..

[41] Thommes M (2015). Physisorption of gases, with special reference to the evaluation of surface area and pore size distribution (IUPAC Technical Report). Pure Appl. Chem..

[42] Rahman MM, Muttakin M, Pal A, Shafiullah AZ, Saha BB (2019). A statistical approach to determine optimal models for IUPAC-classified adsorption isotherms. Energies.

[43] Yang Y (2021). Engineering ruthenium-based electrocatalysts for effective hydrogen evolution reaction. Nano-Micro Lett..

[44] Zhu J, Hu L, Zhao P, Lee LYS, Wong K-Y (2019). Recent advances in electrocatalytic hydrogen evolution using nanoparticles. Chem. Rev..

[45] Murthy AP, Theerthagiri J, Madhavan J (2018). Insights on tafel constant in the analysis of hydrogen evolution reaction. J. Phys. Chem. C.

[46] Sukhbaatar B, Yoon S, Yoo B (2022). Simple synthesis of a CoO nanoparticle-decorated nitrogen-doped carbon catalyst from spent coffee grounds for alkaline hydrogen evolution. J. Mater. Sci..

[47] Li F (2018). Mechanochemically assisted synthesis of a Ru catalyst for hydrogen evolution with performance superior to Pt in both acidic and alkaline media. Adv. Mater..

[48] Fei H (2015). Atomic cobalt on nitrogen-doped graphene for hydrogen generation. Nat. Commun..

[49] Zou X (2014). Cobalt-embedded nitrogen-rich carbon nanotubes efficiently catalyze hydrogen evolution reaction at all pH values. Angew. Chem. Int. Ed. Engl..

[50] Pandey K, Jeong HK (2023). Coffee waste-derived porous carbon for hydrogen and oxygen evolution reaction. Chem. Phys. Impact.

[51] You B (2015). High-performance overall water splitting electrocatalysts derived from cobalt-based metal-organic frameworks. Chem. Mater..

[52] Yuan C-Z (2018). Molecule-assisted synthesis of highly dispersed ultrasmall RuO_2_ nanoparticles on nitrogen-doped carbon matrix as ultraefficient bifunctional electrocatalysts for overall water splitting. ACS Sustain. Chem. Eng..

[53] Huang S (2017). N-, O-, and S-Tridoped carbon-encapsulated Co_9_S_8_ nanomaterials: Efficient bifunctional electrocatalysts for overall water splitting. Adv. Funct. Mater..

[54] Yoon S, Kim J, Lim J-H, Yoo B (2018). Cobalt iron-phosphorus synthesized by electrodeposition as highly active and stable bifunctional catalyst for full water splitting. J. Electrochem. Soc..

[55] Savinova, E. R. & Oshchepkov, A. G. in *Comprehensive Inorganic Chemistry III (Third Edition)* (eds Jan Reedijk & Kenneth R. Poeppelmeier) 492–550 (Elsevier, 2023).

[56] Duan M (2023). Boosting alkaline hydrogen evolution performance by constructing ultrasmall Ru clusters/Na^+^, K^+^-decorated porous carbon composites. Nano Res..

[57] Su P (2021). Exceptional electrochemical HER performance with enhanced electron transfer between Ru nanoparticles and single atoms dispersed on a carbon substrate. Angew. Chem. Int. Ed..

[58] Connor P, Schuch J, Kaiser B, Jaegermann W (2020). The determination of electrochemical active surface area and specific capacity revisited for the system MnO_x_ as an oxygen evolution catalyst. Z. Phys. Chem..

[59] Kibsgaard J, Jaramillo TF (2014). Molybdenum phosphosulfide: An active, acid-stable, earth-abundant catalyst for the hydrogen evolution reaction. Angew. Chem. Int. Ed..

