# Supplementary material for: Uniformly dispersed ruthenium nanoparticles on porous carbon from coffee waste outperform platinum for hydrogen evolution reaction in alkaline media
Source: Sci Rep. 2024 Mar 11;14:5850. doi: 10.1038/s41598-024-56510-7 (PMC10925596; doi:10.1038/s41598-024-56510-7)
Supplement: Supplementary file 1 — Supplementary Information. [file 41598_2024_56510_MOESM1_ESM.docx]

**Supplementary Information**

****Uniformly dispersed ruthenium nanoparticles on porous carbon from coffee waste outperform platinum for hydrogen evolution reaction in alkaline media****

Bayaraa Sukhbaatar ^a^, Wang Qing ^a^, Jinmyeong Seo ^a^, Sanghwa Yoon ^a *^ and Bongyoung Yoo ^a*^

^a^ Department of Materials Science and Chemical Engineering, Hanyang University, Ansan 15588, Korea

**Keywords:** ruthenium nanoparticles, carbon catalyst, hydrogen evolution reaction, spent coffee grounds, HER catalyst

***Corresponding Author:**

*^*^ E-mail:* [*indada@naver.com*](mailto:indada@naver.com) *(Sanghwa Yoon)*

*^*^ E-mail:* [*byyoo@hanyang.ac.kr*](mailto:byyoo@hanyang.ac.kr) *(Bongyoung Yoo)*

1. Synthesis of comparative samples
2. Supplementary Figures
3. Supplementary Tables
4. Supplementary Notes
5. References

**Total number of pages: 19 (S1-S19)**

**Total number of Tables: 3 (Table S1 - Table S3)**

**Total number of Figures: 14 (Figure S1 - Figure S14)**

1. **Synthesis of comparative samples**

**Synthesis of Ru@CB**

Carbon black (Super P® conductive, >99%) was used as carbon support to fabricate Ru@CB. First, 10mg mL^-1^ ruthenium chloride hydrate solution is prepared in ethanol as stock solution. Next, 2.08 mL (equivalent to 0.1mM, 20.74mg) of stock solution is placed into the tube with 2.0 mL ethanol. Then, 100 mg carbon black is added, sonicated for 30 minutes, and agitated for 12 hours under a magnetic stirrer (Ru-impregnated carbon). Finally, the mixture was dried at 110°C, mixed well with mortar and pestle, and annealed under nitrogen gas, flowing 500 mL min^-1^ at 700°C for 2 hours.

**Synthesis of Ru@SCC-Z**

SCC-Z was used as carbon support for the fabrication of Ru@SCC-Z. In the first step, powdered SCG was washed with 1.0M HCl several times to remove impurities and dried. Then, mixed well with ZnCl_2_ (1:1 w/w) in the mortar and pestle, pyrolyzed in the furnace tube with nitrogen gas flowing at 500 mL min^-1^ at 800°C for 2 hours. After cooling down under nitrogen flow, the sample was leached with 1.0 M hydrochloric acid, rinsed with distilled water until neutral, and dried at 105°C for 12 hours, denoted as SCC-Z. Ru@SCC-Z was prepared using the same process as other sample synthesis in the second step.

**Synthesis of Ru@SCC-KU-N3**

SCC-KU was used as carbon support for the fabrication of Ru@SCC-N3. First, 10mg mL^-1^ ruthenium chloride hydrate solution is prepared in ethanol as stock solution. Next, 2.08 mL (equivalent to 0.1 mM, 20.74mg) of stock solution was placed into the tube, and 0.3 mM of 1,10-phenanthroline monohydrate and 2.0 mL of ethanol were added. It mixed well until a brown color complex is formed. Then, 100 mg SCC-KU is added, sonicated for 30 minutes, and agitated for 12 hours under a magnetic stirrer. Finally, the mixture was dried at 110°C, mixed well in the mortar and pestle, and annealed under nitrogen gas, flowing 500 mL min^-1^ at 700°C for 2 hours.

**Synthesis of Ru@SCC-KU-U**

SCC-KU was used as carbon support for the fabrication of Ru@SCC-KU-U. The fabrication process was the same as the Ru@SCC-KU. In the final step, 1.0 g of urea was mixed with Ru-impregnated carbon and mixed well. The mixture was annealed in the same condition.

1. **Supplementary Figures**


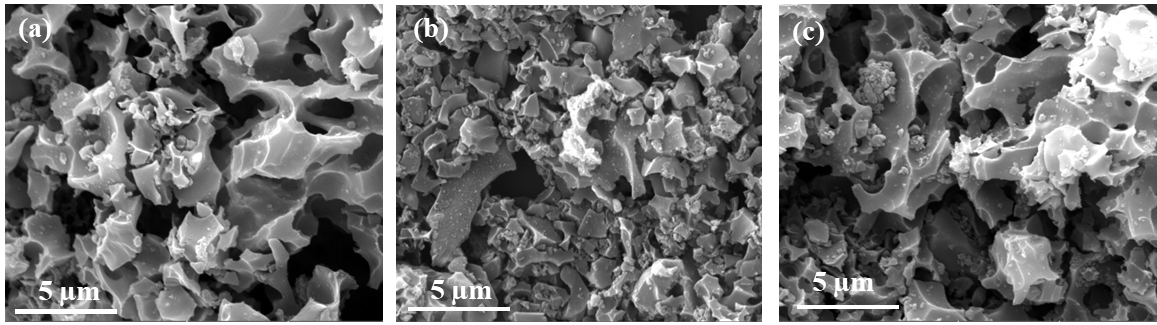


**Fig. S1.** FE-SEM images of (**a**) SCC-KU, (**b**) Ru@SCC-KU-U, and (**c**) Ru@SCC-KU-N3.


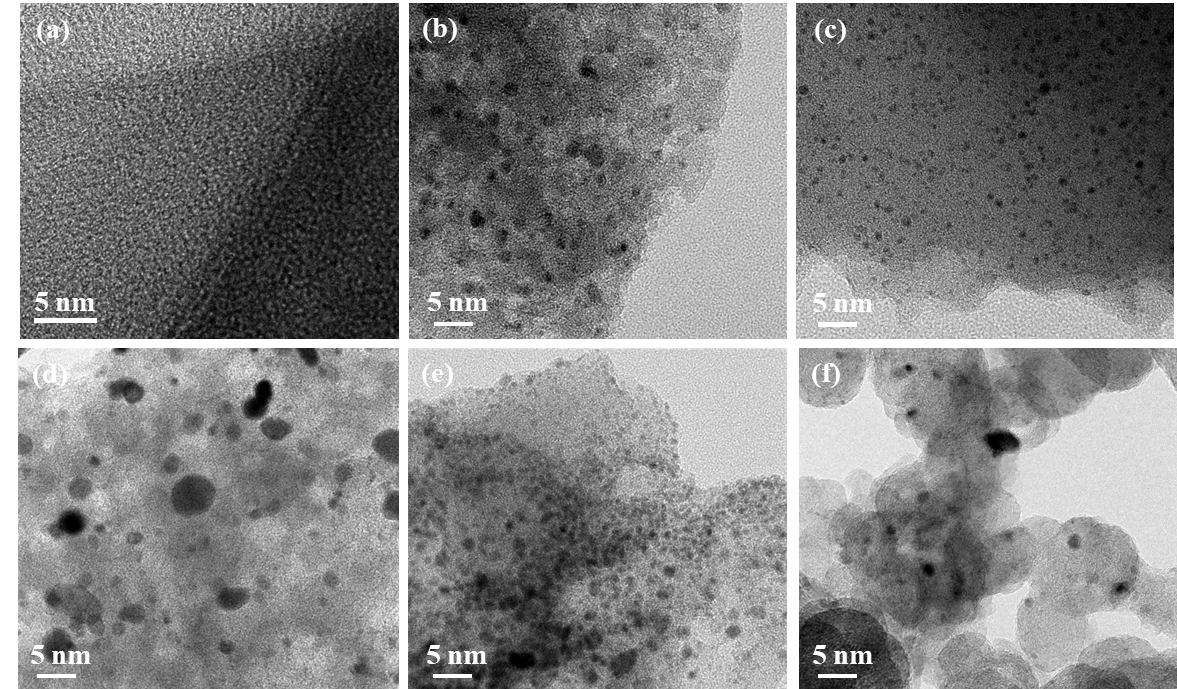


**Fig. S2.** TEM images of (**a**) SCC-KU, (**b**) Ru@SCC-KU-8, (**c**) Ru@SCC-KU-6, (**d**) Ru@SCC-KU-U, (**e**) Ru@SCC-KU-N3, and (**f**) Ru@CB.


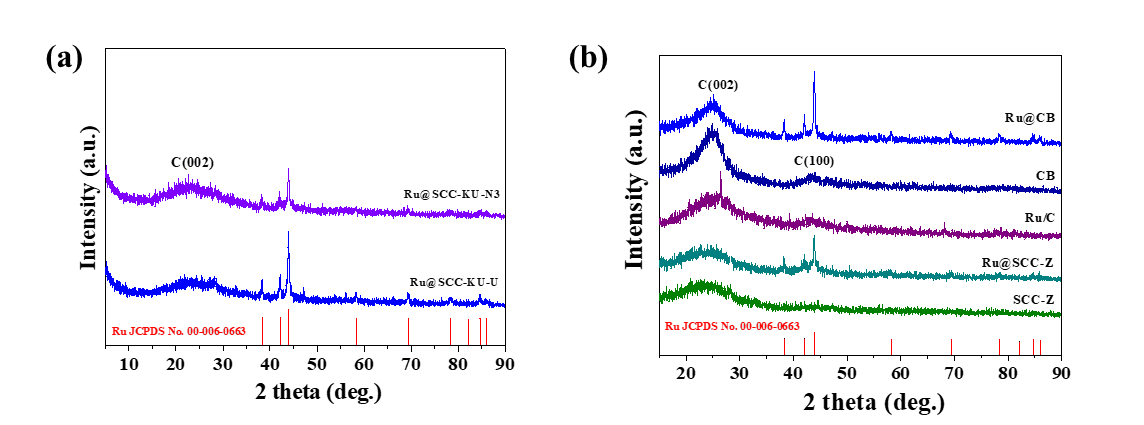


**Fig. S3.** XRD patterns of (**a**) Ru@SCC-KU-N3 and Ru@SCC-KU-U, and (**b**) other comparative catalysts.

**Fig. S4.** Raman spectrum of carbon catalysts.


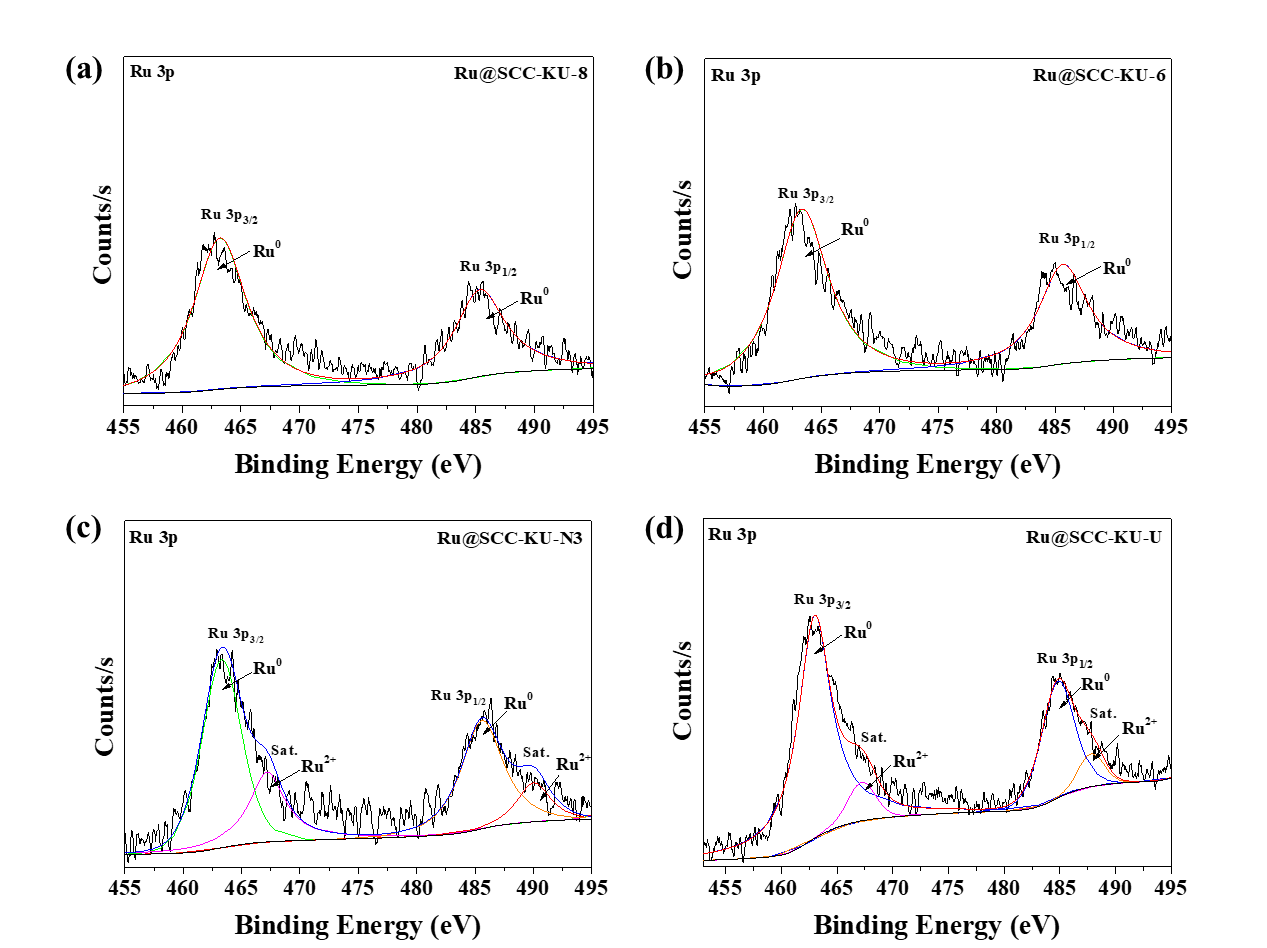


**Fig. S5**. High-resolution XPS spectra of Ru3p of (**a**) Ru@SCC-KU-8, (**b**) Ru@SCC-KU-6, (**c**) RU@SCC-KU-N3, and (**d**) Ru@SCC-KU-N3.

**
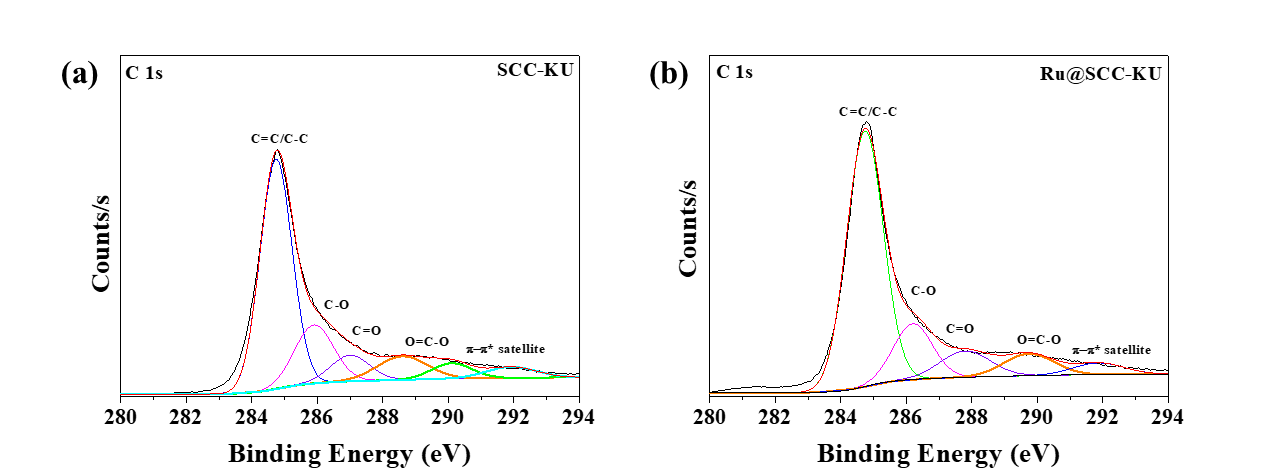
**

**Fig. S6**. The C1s high-resolution XPS spectra of (**a**) SCC-KU and (**b**) Ru@SCC-KU.


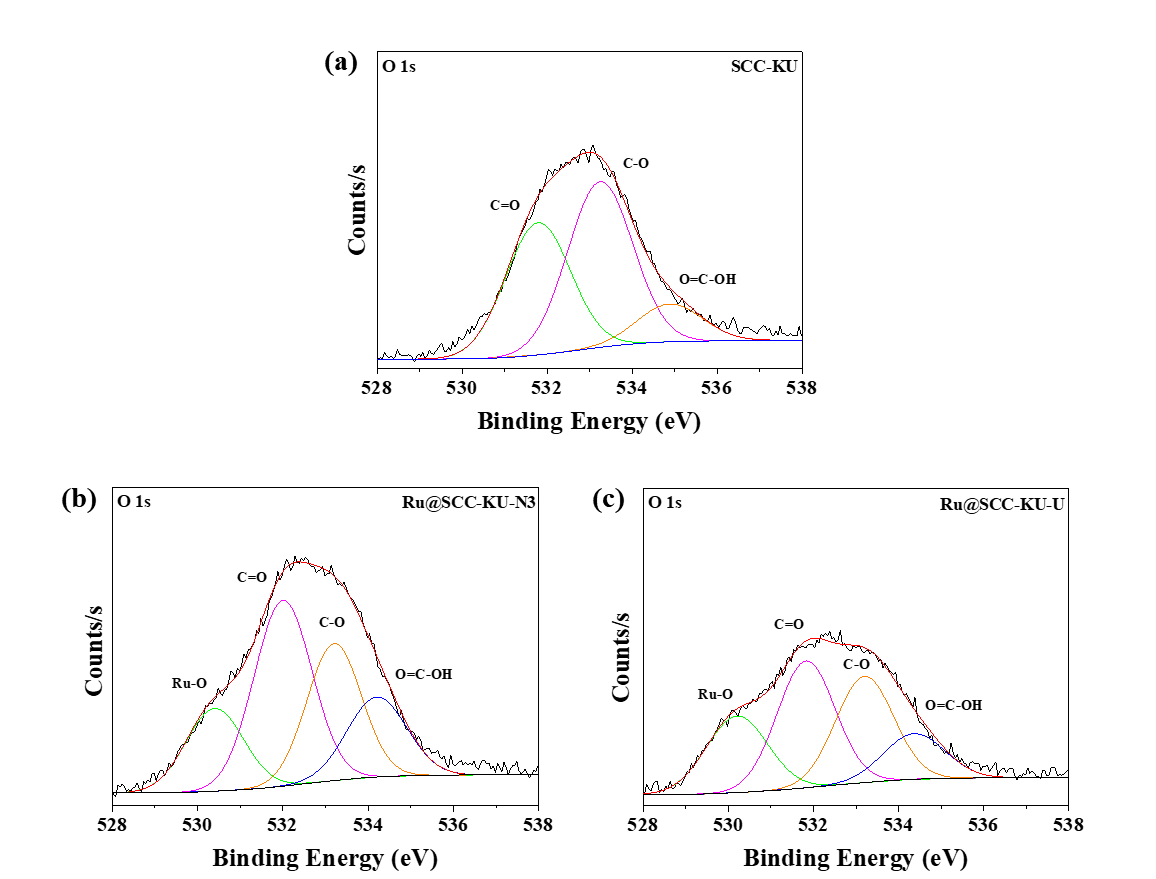


**Fig. S7**. High-resolution XPS spectra of O1s of (**a**) SCC-KU, (**b**) Ru@SCC-KU-N3, and (**c**) Ru@SCC-KU-U.

**Fig. S8.** Calibration of Hg/HgO, NaOH (1.0M) reference electrode in 1.0 M NaOH electrolyte.


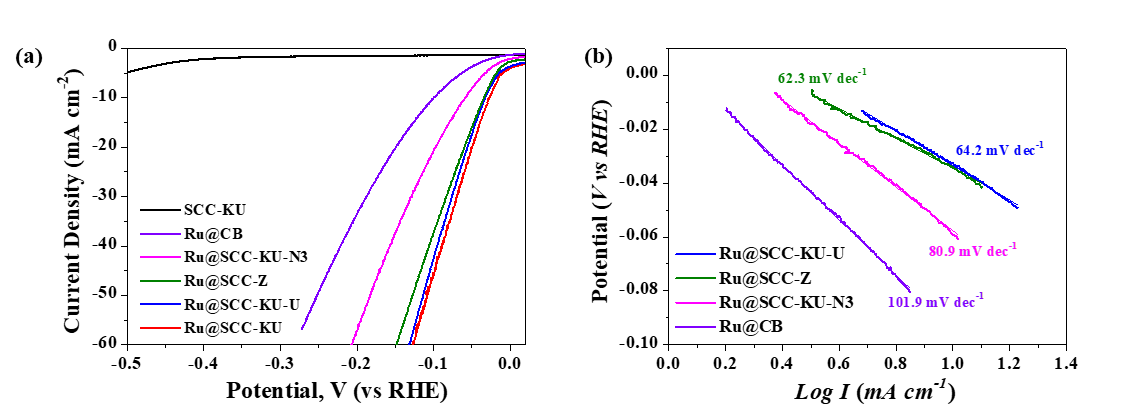


**Fig. S9.** (**a**) Linear sweep voltammetry (LSV) curves of comparative catalysts, (**b**) Tafel curves obtained from LSV of Ru@SCC-KU-U, Ru@SCC-KU-Z, Ru@SCC-KU-N3, and Ru@CB.


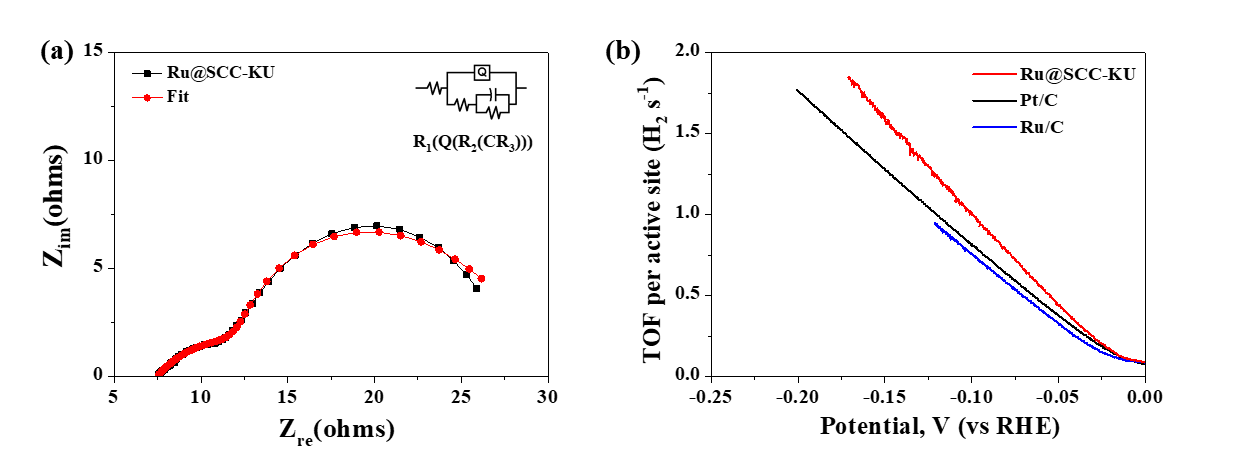


**Fig. S10.** (a) Equivalent circuit fit for EIS data of Ru@SCC-KU. **(b)** TOF values of Ru@SCC-KU, Pt/C, and Ru/C in alkaline electrolyte extracted from LSV polarizations.

**Fig. S11.** LSV curves of EDTA and SCN^-^ poison examination for Ru@SCC-KU in 1.0M NaOH.

**
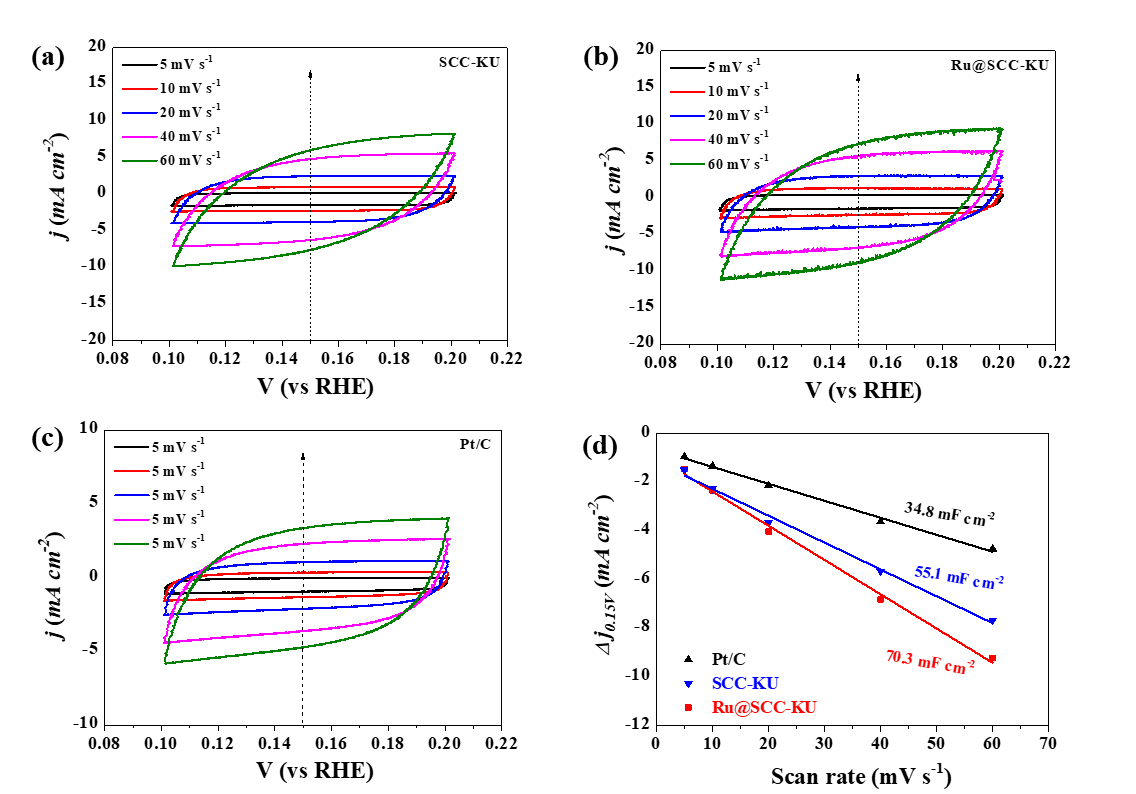
**

**Fig. S12.** The cyclic voltammetry (CV) curves at different scan rates (5, 10, 20, 40, 60 mV s^−1^) at the non-faradaic region (from 0.1 to 0.2 V vs. RHE) for (**a**) SCC-KU, (**b**) Ru@SCC-KU, and (**c**) Pt/C. (**d**) Double-layer capacitance is estimated by anodic current densities at 0.15V vs. RHE as a function of the scan rates.


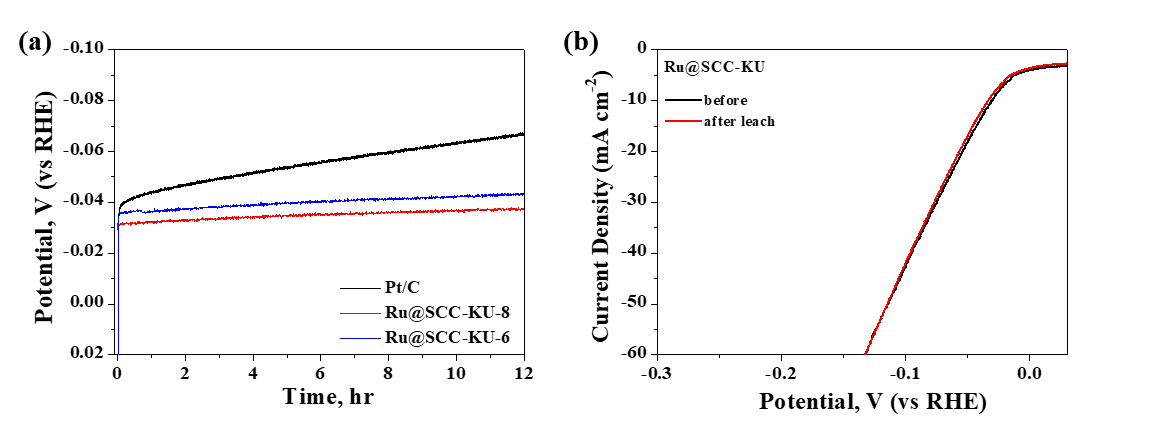


**Fig. S13.** (**a**) Chronopotentionmetric (CP) curves of catalysts were recorded at 10 mA cm^-2^ of current density for 12 hours. (**b**) LSV curves of Ru@SCC-KU, corresponding before and after leaching with 1.0 M HCl.


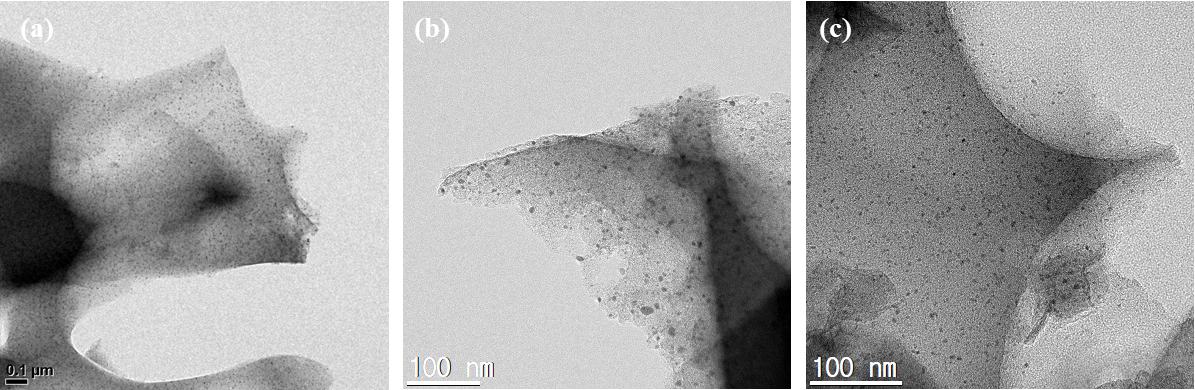


**Fig. S14.** TEM images of (**a**) before, (**b**) after HER CP test for 24 hours, and (**c**) after leaching of Ru@SCC-KU.

1. **Supplementary Tables**

Table S1. Contents of elements of coffee waste-derived carbon catalysts (XPS)

| Contents |  | Atomic, % | |  | | |
| --- | --- | --- | --- | --- | --- | --- |
|  | C1s | N1s | O1s | | Ru3p |  |
| SCC-KU | 92.7 | - | 7.33 | | - |  |
| Ru@SCC-KU | 94.5 | - | 5.05 | | 0.49 |  |
| Ru@SCC-KU-6 | 94.7 | - | 4.8 | | 0.49 |  |
| Ru@SCC-KU-8 | 95.3 | - | 4.19 | | 0.51 |  |
| Ru@SCC-KU-N3 | 87.5 | 2.62 | 9.19 | | 0.72 |  |
| Ru@SCC-KU-U | 89.6 | 1.73 | 7.75 | | 0.93 |  |

Table S2. Pore structure parameters of carbon catalysts

| Samples | Surface area S_BET_ (m^2^ g^-1^) | Average pore size  (Å) | Average pore volume  (cm^3^ g^-1^) |
| --- | --- | --- | --- |
| SCC-KU | 1243.9 | 14.7 | 0.36 |
| Ru@SCC-KU | 1170.9 | 16.2 | 0.42 |
| Ru@SCC-KU-8 | 1318.6 | 17.7 | 0.53 |
| Ru@SCC-KU-6 | 1263.3 | 18.2 | 0.54 |
| Ru@SCG-KU-N3 | 599.8 | 14.8 | 0.17 |
| Ru@SCG-KU-U | 1452.1 | 16.3 | 0.54 |

**Table S3.** Summary of HER performance overpotentials of carbon-based electrocatalysts in alkaline electrolyte

| Catalysts | Electrolyte | Overpotential  @ 10 mA cm^-2^ | Tafel slope  (mV dec^-1^) | Ref |
| --- | --- | --- | --- | --- |
| Ru@MWCNT | 1.0 M KOH | 14 mV | 27.0 | ^1^ |
| Ru/rGo-700 | 1.0 M KOH | 26 mV | 34.7 | ^2^ |
| RuO_2_/N-C | 1.0 M KOH | 40 mV | 44.0 | ^3^ |
| Ru/NC | 1.0 M KOH | 14.8 mV | 22.3 | ^4^ |
| Ru@CQDs | 1.0 M KOH | 65 mV | 63 | *^5^* |
| Ru@NC | 1.0 M KOH | 39 mV | 37.9 | ^6^ |
| Co-P/NC | 1.0 M KOH | 191 mV | 51 | ^7^ |
| Co-NRCNTs | 1.0 M KOH | 370 mV | - | ^8^ |
| Co_9_S_8_@NOSC (N,O,S-doped carbon) | 1.0 M KOH | 320 mV | - | ^9^ |
| Co-NG (Cobalt SAC on N-Graphene) | 1.0 M NaOH | 270 mV | - | ^10^ |
| CCW-KOH-600 (Carbonized coffee waste) | 6.0 M KOH | 210 mV | 120 | ^11^ |
| Ru@SCC-KU-N3 | 1.0 M NaOH | 62.4 mV | 80.4 | **This work** |
| Ru-SCC-KU | 1.0 M NaOH | **27.0 mV** | **58.4** | **This work** |

1. **Supplementary Notes**

**Supplementary Note 1: Calibration of the reference electrode**

Hg/HgO, NaOH (1.0 M) reference electrode was calibrated concerning the reversible hydrogen electrode (RHE). The H_2_ saturated electrolyte with Pt wires as both the working and counter electrodes were used in the calibration experiment. Cyclo voltammetry measurement was achieved at a 1 mV s^-1^ scan rate. We determined an average of two anodic and cathodic potentials that scan crossed zero current for the hydrogen electrode reaction. According to the results shown in **Fig. S8**, *E*(RHE) = E (Hg/HgO) + 0.926 V.

**Supplementary Note 2: Turnover frequency (TOF) calculations**

To calculate the per-site turnover frequency (TOF) of Ru@SCC-KU catalysts, we used the following equations ^12^:

$TOF (H_{2}/s)=\frac{\mathrm{Total}H_{2} turnovers per geometric area}{active sites per geometric area}$ (1)

The total hydrogen turnover can be calculated from the value of current density obtained from LSV curves as follows:

$\mathrm{Total}H_{2} turnovers=\frac{6.022\cdot{10}^{23} {mol}^{-1}}{2\cdot96500 C {mol}^{-1}}\times\left| j \right|\frac{mA}{{cm}^{2}}=3.12\cdot{10}^{15} \frac{H_{2}}{{s\cdot cm}^{2}} per \frac{mA}{{cm}^{2}}$ (2)

$${active sites}_{Ru@SCC-KU}=\frac{catalyst mass loading \times Ru\% \times6.022\cdot{10}^{23} {mol}^{-1}}{molar mass of Ru}$$

$= \frac{0.612 \times\cdot{10}^{-3} g {cm}^{2} \times3.9 wt\% \times6.022\cdot{10}^{23} {mol}^{-1}}{101.07 g {mol}^{-1}}= 1.42\cdot{10}^{17} Ru sites per {cm}^{2}$ (3)

$\mathrm{TOF}_{Ru@SCC-KU} \left( H_{2} s^{-1} \right)=\frac{3.12\cdot{10}^{15}}{1.42\cdot{10}^{17}}\times\left| j \right|=0.022\times\left| j \right|$ (4)

**Supplementary Note 3: Calculation of electrochemical active surface area (ECSA)**

To obtain double layer capacitance (*C*_dl_), the CV was carried out with the potential between 0.10 to 0.20 V vs. RHE at different scan rates (5, 10, 20, 40, and 60 mV/s), as shown in **Figure S5**. The differences in current density variation (∆j) at a potential of 0.15 V vs. RHE will be plotted against the scan rate. The plotted lines can be fitted by linear regression, where the slope is twice *C*_dl_. The *C*_dl_ values for SCC-KU, Ru@SCC-KU, and Pt/C are calculated to be 55.1, 70.3, and 34.8 mF/cm^2^, respectively. The specific capacitance can be converted into an ECSA using the specific capacitance value for a flat standard with 1 cm^2^ of actual surface area. As reported references, the specific capacitance of carbon electrode materials is usually considered to be 20.9 μF/cm^2^ to calculate the ECSA ^13^, and calculated according to Equation ^14^ (5):

$ECSA=\frac{C_{\mathrm{dl}}}{20.9 \mu F\cdot\mathrm{cm}^{-2}\cdot per \mathrm{cm}_{\mathrm{ECSA}}^{2}}\mathrm{cm}_{\mathrm{ECSA}}^{2}$ (5)

For Ru@SCC-KU: $ECSA=\frac{70.3 mF\cdot\mathrm{cm}^{-2}}{20.9 \mu F\cdot\mathrm{cm}^{-2}\cdot per \mathrm{cm}_{\mathrm{ECSA}}^{2}}{= 3363.6 cm}_{\mathrm{ECSA}}^{2}$ (6)

For SCC-KU: $ECSA=\frac{55.1 mF\cdot\mathrm{cm}^{-2}}{20.9 \mu F\cdot\mathrm{cm}^{-2}\cdot per \mathrm{cm}_{\mathrm{ECSA}}^{2}}{= 2636.4 cm}_{\mathrm{ECSA}}^{2}$ (7)

For Pt/C: $ECSA=\frac{34.8 mF\cdot\mathrm{cm}^{-2}}{20.9 \mu F\cdot\mathrm{cm}^{-2}\cdot per \mathrm{cm}_{\mathrm{ECSA}}^{2}}{= 1665.1 cm}_{\mathrm{ECSA}}^{2}$ (8)

If we divide the ECSA by the loading density of Ru centers on the electrode, we can get the averaged area to find one Ru center ^10^:

$A_{ECSA per site}=\frac{ECSA}{active sites}=\frac{{3363.6 cm}_{\mathrm{ECSA}}^{2}}{1.42\cdot{10}^{17}Ru sites per {cm}^{2}}{= 2.3\cdot{10}^{-14}\mathrm{cm}}_{\mathrm{ECSA}}^{2} per Ru$ (9)

$Active sites density \left( sites {cm}^{2} \right)=\frac{1}{A_{ECSA per site}}=4.34\cdot{10}^{13} sites{cm}^{2}$ (10)

1. **References**

1 Kweon, D. H. *et al.* Ruthenium anchored on carbon nanotube electrocatalyst for hydrogen production with enhanced Faradaic efficiency. *Nature communications* **11**, 1278 (2020).

2 Yang, Y. *et al.* Bridge the activity and durability of Ruthenium for hydrogen evolution reaction with the RuOC link. *Chemical Engineering Journal* **433**, 134421 (2022).

3 Yuan, C.-Z. *et al.* Molecule-Assisted Synthesis of Highly Dispersed Ultrasmall RuO2 Nanoparticles on Nitrogen-Doped Carbon Matrix as Ultraefficient Bifunctional Electrocatalysts for Overall Water Splitting. *ACS Sustainable Chemistry & Engineering* **6**, 11529-11535, doi:10.1021/acssuschemeng.8b01709 (2018).

4 He, Q. *et al.* Synergic reaction kinetics over adjacent ruthenium sites for superb hydrogen generation in alkaline media. *Advanced Materials* **34**, 2110604 (2022).

5 Li, W. *et al.* Carbon quantum dots enhanced the activity for the hydrogen evolution reaction in ruthenium-based electrocatalysts. *Materials Chemistry Frontiers* **4**, 277-284, doi:10.1039/C9QM00618D (2020).

6 Ma, E. *et al.* Chitin Derived Carbon Anchored Ultrafine Ru Nanoparticles for Efficient Hydrogen Evolution Reaction. *ACS Sustainable Chemistry & Engineering* **10**, 15530-15537 (2022).

7 You, B. *et al.* High-Performance Overall Water Splitting Electrocatalysts Derived from Cobalt-Based Metal–Organic Frameworks. *Chemistry of Materials* **27**, 7636-7642, doi:10.1021/acs.chemmater.5b02877 (2015).

8 Zou, X. *et al.* Cobalt-Embedded Nitrogen-Rich Carbon Nanotubes Efficiently Catalyze Hydrogen Evolution Reaction at All pH Values. *Angewandte Chemie International Edition* **53**, 4372-4376, doi:<https://doi.org/10.1002/anie.201311111> (2014).

9 Huang, S. *et al.* N-, O-, and S-Tridoped Carbon-Encapsulated Co9S8 Nanomaterials: Efficient Bifunctional Electrocatalysts for Overall Water Splitting. *Advanced Functional Materials* **27**, 1606585, doi:<https://doi.org/10.1002/adfm.201606585> (2017).

10 Fei, H. *et al.* Atomic cobalt on nitrogen-doped graphene for hydrogen generation. *Nature Communications* **6**, 8668, doi:10.1038/ncomms9668 (2015).

11 Pandey, K. & Jeong, H. K. Coffee waste-derived porous carbon for hydrogen and oxygen evolution reaction. *Chemical Physics Impact* **6**, 100175, doi:<https://doi.org/10.1016/j.chphi.2023.100175> (2023).

12 Benck, J. D., Chen, Z., Kuritzky, L. Y., Forman, A. J. & Jaramillo, T. F. Amorphous Molybdenum Sulfide Catalysts for Electrochemical Hydrogen Production: Insights into the Origin of their Catalytic Activity. *ACS Catalysis* **2**, 1916-1923, doi:10.1021/cs300451q (2012).

13 Kibsgaard, J. & Jaramillo, T. F. Molybdenum Phosphosulfide: An Active, Acid-Stable, Earth-Abundant Catalyst for the Hydrogen Evolution Reaction. *Angewandte Chemie International Edition* **53**, 14433-14437, doi:<https://doi.org/10.1002/anie.201408222> (2014).

14 Connor, P., Schuch, J., Kaiser, B. & Jaegermann, W. The Determination of Electrochemical Active Surface Area and Specific Capacity Revisited for the System MnOx as an Oxygen Evolution Catalyst. *Zeitschrift für Physikalische Chemie* **234**, 979-994, doi:doi:10.1515/zpch-2019-1514 (2020).
